# The everyday experience of living with and managing a neurological condition (the LINC study): study design

**DOI:** 10.1186/1471-2377-13-30

**Published:** 2013-03-21

**Authors:** Joan Versnel, Tanya Packer, Lori E Weeks, Jocelyn Brown, Marshall Godwin, Susan Hutchinson, George Kephart, Diane MacKenzie, Kerstin Roger, Robin Stadnyk, Michelle Villeneuve, Grace Warner

**Affiliations:** 1 School of Occupational Therapy, Dalhousie University, 5869 University Avenue, 215 Forrest Building, PO Box 15000, Halifax, NS B3H 4R2, Canada; 2 Department of Applied Human Sciences, University of Prince Edward Island, 550 University Avenue, Charlottetown, PE C1A 4P3, Canada; 3 Discipline of Family Medicine, Faculty of Medicine, Memorial University of Newfoundland, 2420 Health Sciences Centre, St. John’s, NL, A1B 3V6, Canada; 4 Schoolof Health and Human Performance, Stairs House, 6230 South Street, Dalhousie University, PO Box 15000, Halifax, NS B3H 4R2, Canada; 5 Department of Community Health and Epidemiology, Faculty of Medicine, Dalhousie University, Centre for Clinical Research, 5790 University Avenue, Halifax, NS B3H 1V7, Canada; 6 Department of Family Social Sciences, University of Manitoba, 204 Human Ecology Building, Winnipeg, MB, R3T 2N2, Canada; 7 School of Rehabilitation Therapy, Queen’s University, Louise D. Acton Building,31 George Street, Kingston, Ontario, K7L 3N6, Canada

**Keywords:** Participation framework, Self-management, Chronic disease, Services, Children, Youth adults, Parents, Disability

## Abstract

**Background:**

The impact of neurological conditions on individuals, families and society is increasing and having a significant economic impact in Canada. While some economic data is known, the human costs of living with a neurological condition are poorly understood and rarely factored into future burden analyses. The “Living with the Impact of a Neurological Condition (LINC)” study aims to fill this gap. It seeks to understand, for children and adults with neurological conditions, the supports and resources that make everyday life possible and meaningful.

**Methods/design:**

The LINC study is a nested study using mixed methods. We are interested in the following outcomes specifically: health status; resource utilization; self-management strategies; and participation. Three studies captured data from multiple sources, in multiple ways and from multiple perspectives. Study One: a population-based survey of adults (n = 1500), aged 17 and over and parents (n = 200) of children aged 5 to 16 with a neurological condition. Study Two: a prospective cohort study of 140 adults and parents carried out using monthly telephone calls for 10 months; and Study Three: a multiple perspective case study (MPCS) of 12 adults and 6 parents of children with a neurological condition. For those individuals who participate in the MPCS, we will have data from all three studies giving us rich, in depth insights into their daily lives and how they cope with barriers to living in meaningful ways.

**Discussion:**

The LINC study will collect, for the first time in Canada, data that reflects the impact of living with a neurological condition from the perspectives of the individuals themselves. A variety of tools will be used in a combination, which is unique and innovative. This study will highlight the commonalities of burden that Canadians living with neurological conditions experience as well as their strategies for managing everyday life.

## Background

There is a need to understand the severity of disability experienced by adults and parents of children living with neurological conditions in Canada. Results of the Participation and Activity Limitation Survey (PALS) [1] indicated that while 16.6% of the adults in Canada reported a disability in 2006, it is not known how many of these 4.2 million adults are living with a disability as a result of a neurological condition. Results of this study also showed that 1.46 million people aged 15–64 reported needing help with daily activities, with almost half of these reporting not receiving the help they need.

The Neurological Health Charities of Canada (NHCC) [2] report that the impact of neurological conditions on individuals, families and societies is staggering, and these impacts will increase, as neurological conditions become the leading causes of death and disability in Canada in the next twenty years. The Canadian Institute for Health Information (CIHI) [3] found that neurological diseases, disorders and injuries have a significant economic impact on Canada, costing 8.8 billion dollars in 2000–2001, with indirect costs (e.g. lost productivity due to long-term disability or premature death) accounting for an estimated $6.5 billion compared with $2.3 billion in direct healthcare costs (e.g., hospital care, physician care, and drugs). In 2004-2005, 9% of acute care hospitalization and 19% of hospital stays in Canada were associated with neurological conditions as primary or secondary diagnoses [3].While economic costs are great, information regarding health care utilization and the economic burden of neurological conditions does not explicate the human costs of living with a neurological condition.

While there has been some attention to the “illness experience” of persons with a variety of specific neurological conditions [4-10], very few studies have examined the commonality of those experiences. For example, shared experiences appear to include disrupted relationships and a reduction in participation in personally meaningful activities (e.g., employment, shared family activities) [1]. While these impacts are reported in the diagnosis specific literature, few studies examine similarities and differences across diagnoses. Moreover, the ways in which individuals and parents of children with neurological conditions are supported and the experience of receiving support is not well described in the current literature.

### The LINC Study

The LINC study seeks to fill these gaps: to understand the everyday lives of people with neurological conditions who strive to work, go to school, raise families, and participate in the community. It seeks to understand, for children and adults with neurological conditions, the support and resources that make everyday life possible and meaningful.

The LINC study is based on the assumptions that 1) individuals with neurological conditions wish to live meaningful and productive lives; 2) management of the impact on everyday life is not diagnosis specific; 3) management is a partnership between individuals/families and service providers; and 4) solutions to everyday problems are dependent on seamless integration of health, social and community services.

**Figure 1:**
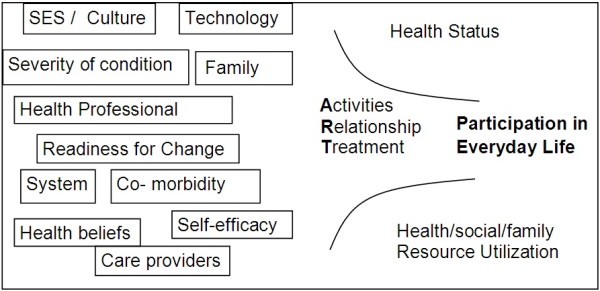
pARTicipation Model.

The study has explicitly adopted a self-management focus, reflecting current best practice in chronic condition management [11]. More specifically, the LINC study is guided by the pARTicipation Framework (Figure 1) [12]. This Framework recognizes the seminal work of Corbin and Strauss [13] who identified three forms of “work” required when managing life with any chronic condition. In the pARTicipation Framework, these forms of work have been relabeled the ART of self-management: management of daily activities (**A**ctivities), emotions and relationships (**R**elationships) and the condition itself (**T**reatment). Literature shows that successful management is influenced by many predictive and mediating factors (e.g. family support, co-morbidity, economic status) [14-16]. The World Health Organization’s International Classification of Function (ICF) acknowledges the complexity of participation and was used to help define the important variable of everyday participation [17].

The overall aims of the LINC study are to explore:

1. The impact of a neurological condition on the everyday life experiences of Canadians, including parents of children with neurological conditions;

2. The complex inter-dependence between children and/or adults with a neurological condition and their families; and

3. The ability of health, social and community services and agencies to support individuals andfamilies to self-manage life with a neurological condition.

Outcomes of interest in the LINC study and their importance to different stakeholders are:

1. Participation in everyday activities, of primary importance to people with neurological conditions and their families;

2. Health/social/community resource utilization (including family, health, social, and community resources), of interest to governments, service providers and communities; and

3. Health status (depression, physical status etc.), important to individuals and often the stated goals of health providers.

## Methods/design

The LINC study is a nested study using mixed methods. A variety of strategies are used in order to capture data from multiple sources, in multiple ways and from multiple perspectives. The nested approach successively draws participants for each study from those in the previous study. The three studies, with projected sample size are: 1) a population-based survey of adults (n = 1500) age 17 and older who have a neurological condition and parents (n = 200) of children age 5–16 who have a neurological condition, 2) a cohort study (n = 140) adults and parents of children with neurological conditions, and 3) a multiple perspective case study (MPCS) of 12 adults and 6 parents of children with a neurological condition.

### Ethics approval

For all components of this study, ethical approval was received from the Health Canada and Public Health Agency of Canada Research Ethics Board as well as the appropriate ethics review boards at Dalhousie University, Queens University, the University of Manitoba, and the University of Prince Edward Island. Data collection in Newfoundland and Labrador was acknowledged by the provincial Health Research Ethics Authority. Relevant information regarding the study purpose, design, risks and use of the data is provided to participants prior to participation. Completion of the population survey is considered implied consent. Provision of an e-mail address is requested in order for participants to be able to take a break and return to the survey. Although requested, the email address is not required, making anonymous participation possible. On completion of the survey, participants are invited to express interest in other research studies including the cohort study. Participants replying in affirmative are requested to provide contact details. Oral consent is required to participate in the cohort study and signed informed consent is required for participation in the MPCS.

### Population-based survey: *a snapshot in time*

The population-based survey will provide a “snapshot in time” by collecting data from a large sample of adults with neurological conditions and parents of children with neurological conditions at one point in time. The primary purpose is to describe the impact of a neurological condition on participation in everyday life and, where possible, using items common to routine Statistics Canada surveys, compare results to the full Canadian population. The primary outcomes of interest are participation, health status and resource utilization. In addition, use of self-management behaviors is being measured. While the primary mode of data collection is an online self-report survey, we recognize that some people with neurological conditions are better able to respond verbally or via paper and pencil, hence these options are also provided.

### Participants

Adults age 17 and older living with a neurological condition in Canada, and any parent of a child or children age 5–16 with any neurological condition living in Canada, are eligible to participate. The breadth of neurological disorders included in this research are: neurotrauma (e.g., acquired brain injury, brain tumor, spinal cord injury, hydrocephalus); neuromuscular disorders (e.g., cerebral palsy, epilepsy and spina bifida); degenerative demylenating conditions (e.g., multiple sclerosis, or Guillian-Barre syndrome); dystonia, Tourette syndrome, and movement and other neurodegenerative disorders (e.g., Parkinson’s disease, Huntington’s disease, muscular dystrophies, dementia and ALS [Lou Gehrig’s disease]). The survey is available in English and French.

### Measurement tools

Based on the pARTicipation model, the outcomes of interest are: participation, health status and resource utilization. Potential predictive variables (e.g. health professional support, social support, health beliefs, and demographic data) are being measured. Demographic data include age, gender, postal code, educational attainment, socio-economic and employment status.

There is no single validated tool available to describe the complex impact of a neurological condition on participation in everyday activities or to describe use of health, social and family resources. To overcome these difficulties, measurement tools include well-validated, psychometrically robust surveys typically designed for research studies and items/modules drawn from Statistics Canada surveys, such as the PALS, the General Social Survey (GSS) the Canadian Community Health Survey (CCHS) designed for routine population level data collection. The use of Statistics Canada modules has the potential benefit of comparison to existing data drawn from routine census data. The data collection tools are listed in Table 1.

**Table 1:** Summary of the instruments used by variable category in the population-based survey

**Tool**	**Population study**			**Cohort study**			
	Adult*	**Parent**	**Parent report on child**	**Adult**	**Parent**	**Parent report on child**	**Mature minor**
**Diagnosis and Co-morbidity**							
CCHS/ Survey of Living with a Neurological Condition (SLNCC) – Chronic Conditions [18,19]	✓	✓	✓				
SLNCC – Diagnosis [19]	✓	✓	✓				
**Participation**							
Assessment of Life Habits (Life - H) [20,21]				✓		✓	
Pediatric Quality of Life (PedsQL) Measurement Model - Family Impact Module [22]		✓					
Quality of Life Outcomes in Neurological Disorders (Neuro-QOL): Satisfaction with Social Roles and Activities Module, Ability to Participate Module [23]	✓			✓			
CCHS - voluntary organization participation [18]	✓	✓	✓				
CCHS – Activities of Daily Living Module: Loss of Productivity Module; Recent Life Events; Restriction of Activities Module; Voluntary organization participation [18]							
GSS – Two Week Disability Module Occupation and Health [24]	✓	✓	✓	✓	✓	✓	
PALS –“Filter Questions” Module: Labour Force Discrimination; Local Transportation [25]	✓		✓				
SLNCC – Restriction of activities Module [19]	✓		✓	✓	✓		
**Resource Utilization**							
Multidimensional Scale of Perceived Social Support (MSPSS) [26,27]							
CCHS – Access to health care services: Contacts with Health Professionals; Health Care Utilization Module; Patient Satisfaction, Community Based Care; Patient Satisfaction, Health Care Services [18]							
PALS –Education Modification: Employment Modification; Housing Module; Leisure and Recreation [25]							
SLNCC – Formal assistance: Informal assistance; Out of pocket expenses; Medication use [19]							
**Self-management of Condition and Everyday Life**							
Giving Youth a Voice (GYV): (young adults: 17-26 only) [28]	✓			✓			✓
Lorig Self-Efficacy [29]	✓	✓	✓				
Partners in Health (PIH) [30]	✓						
Patient Activation Measure (PAM) [31]	✓	✓	✓				✓
Patient Assessment of Chronic Illness Care (PACIC) [32]				✓	✓		✓
Simple Lifestyle Indicator Questionnaire (SLIQ) [33]	✓	✓					
Transition Readiness Assessment Questionnaire (TRAQ) [34]	✓		✓				✓
CCHS: Coping with Stress Module; Spiritual Values [18]	✓	✓					
**Health Status**							
Health Utility Index (HUI) 3 [35,36]	✓	✓	✓				
Neuro-QOL Domains: Upper Extremity Function; Lower Extremity Function; Fatigue; Sleep Disturbance; Communication; Applied Cognition-General Concerns; Applied Cognition-Executive Function; Emotional & Behavioural Dyscontrol; Depression; Anxiety; Positive Affect and Well-being [23]	✓						
PedsQL Modules: Physical Function; Emotional Function; School Function; Social Function [37]			✓				
Short-Form Health Status (SF-36) [38]	✓	✓					✓
CCHS – General Health Module [18]	✓	✓	✓		✓	✓	✓
GSS – Health Status [24]				✓	✓	✓	✓
**Demographic Information**							
CCHS: Education; Socio-demographic characteristics [18,19]	✓	✓	✓				
CCHS/SLNCC – Income [18,19]	✓	✓					
CCHS/SLNCC – Labour force [18,19]	✓	✓	✓				
PALS/SLNCC – Employment Status [18,25]	✓	✓	✓				

* All respondents provide self-report data about themselves unless otherwise specified.

### Variables and measures

#### Pilot study

The online survey was pilot tested first by investigators, research staff and members of the Project Advisory Committee. This was followed by pilot testing with adults and parents with neurological conditions. After receiving recommendations from those who participated in the pilot study, we made the following modifications to the protocol and letter of invitation. Additional details were provided about the LINC study and the relationship between the various components of the study. We specified that participants had to be living in Canada. The time required to complete the survey was changed from 60 minutes to 60–90 minutes. On the advice of pilot study participants with neurological conditions, text boxes were added for participants to further explain the reason behind choosing their response or to discuss other pertinent issues not captured by the survey items.

#### Translation

Where French language translations were available, including some validated tools and all Statistics Canada modules, these were used. Any new items without French translation were first translated from English into French (henceforth referred to as 'forward translation’) by a translator whose first language is French. The French items produced by the translator were then sent to a second translator to translate the items from French to English (henceforth referred to as ‘back translation’) by a translator whose first language is English. Both translators are registered with the Association of Translators and Interpreters of Nova Scotia. A third individual, a bilingual health professional familiar with the tools, reconciled the documents to determine any discrepancies between the original English document and the English document resulting from the back translation. If discrepancies occurred, the reconciler returned to the original documents to determine if the mistranslation occurred in the French or the English translation. If it occurred in the forward translation, it was returned to the translator for clarification. If it occurred in the back translation, it was determined to be a difference in styling by the back translator and the French translation was retained.

#### Participant recruitment

In order to recruit our target sample of 1500 participants, we utilized several recruitment strategies to maximize reach and representativeness.

1. The NHCC is facilitating recruitment via its member organizations and a data base of individuals interested in research. The NHCC is a national umbrella group with a growing membership. At the commencement of the study it included eighteen national and three provincial organizations. The NHCC member organizations provide the most extensive, single listing of individuals living in the community who have a neurological condition. Recruitment is via a letter of invitation and a poster distributed by NHCC. The NHCC also has a link to the study on its web page and continues to “tweet” frequently about the study.

2. A poster and a letter of invitation are being circulated to organizations, consumer groups, and people with a neurological condition not affiliated with the NHCC, and to any individuals who express interest in the study.

3. The poster and letter of invitation are being circulated via known databases and registries of people with neurological conditions, or practitioners who work with people with neurological conditions.

4. Snowball sampling is occurring via participants forwarding information to friends and family with neurological conditions.

5. The recruitment poster and a link to the survey have been placed on the webpages, Facebook pages and/or other electronic interfaces of Dalhousie University School of Occupational Therapy, and other Universities affiliated with the research team.

6. When invited, members of the research team provide short presentations to consumer groups about the study and how to participate. They also speak on TV and radio about the study.

7. In the case of low numbers or obvious under-representation (e.g. age, diagnosis), targeted recruitment of organizations is being undertaken via emails, telephone calls, talks to stakeholders and professional visits.

8. Finally, the recruitment poster has been placed on community bulletin boards.

#### Data collection procedures

Data are collected via self-report survey, primarily via an online version; a paper and pencil format or telephone interview is available depending on the preference of respondents. Both French and English versions are available. Telephone interviews are conducted by trained research assistants. Respondents who choose this option are made aware of the conditions of confidentiality imposed on the assisting party. Parents of children age 5–16 with a neurological condition complete the parent version of the survey on their own behalf, providing information about their child’s health and the impact of their child’s condition on their own participation, health and health care utilization. Parents who have more than one child with a neurological condition are able provide information about all children with a neurological condition.

The survey has a completion time of between 60 and 90 minutes. Due to the length of the survey, the on-line survey is designed so that participants can exit the survey at any time and re-enter the site later. Reminders are sent to online participants by e-mail if they exit the survey and do not return to it within 7 days. A mailed reminder is sent to those who choose the mailed paper and pencil survey and do not return to it within 21 days. For both methods of data collection, an initial reminder is sent followed by two reminders at two week intervals. The survey will be available for nine months.

#### Data analysis

Data will be analyzed separately for parents and adults with neurological conditions. Analysis of results will provide insight into the extent (proportions and variability) of the impact of living with a neurological condition on the outcomes of interest. As a first step, analyses will be undertaken to detect biases due to the data collection method and to assess the generalizability of results. The data will be examined for missing values to assess completeness. The distribution of all interval data will be plotted to determine whether parametric analyses are appropriate and scatter plots will look for response clusters indicative of systematic biases. Percentages, ranges, medians, means and standard deviations of all variables will be reported.

For items taken from Statistics Canada surveys, data will be compared to national data sets using the appropriate statistical tests for categorical and ordinal variables. Data sets from Statistics Canada surveys will be accessed through the Atlantic Regional Data Centre (ARDC). The ARDC provides secure access to Statistics Canada micro-level survey data for research purposes in a secure setting. Sub-group descriptions will include age, gender, geography (rural versus urban – using postal codes), type and characteristics of condition (episodic, age at onset, deteriorating). Parent data will be analyzed for children age 5–12 and 13–17, and adult data will be partitioned to examine young adults age 17–24, those of working age and those retired. Other sub-group analyses will depend on the variability of the data collected.

#### Cohort study: *A year in the life of people with a neurological condition living in Canada*

The cohort study uses a prospective, longitudinal design to add depth to the knowledge gained in the population-based survey. Data are collected during monthly telephone interviews that include semi-structured and open ended questions, augmented with standardized surveys (conducted either by phone or online). The specific objectives of the cohort study are to: 1) describe in greater detail the impact of a neurological condition on participation, resource utilization and health status; 2) describe the stability versus change in the variables over an 11 month period; and 3) examine if and how variables such as health status, self-efficacy, health care support, social support and self-management skills have an impact on peoples’ participation in everyday activities.

#### Participants

The participants in the cohort study are a sample of convenience, drawn sequentially from participants in the population survey who, on completion, expressed interest in participating in future research. There are three groups of participants:

1. Adults with a neurological condition aged between 17–65 years

2. Parents of children aged 5–16 years with a neurological disorder; and

3. Mature minors aged 13–16 with a neurological condition and whose parent is participating in the cohort study.

Inclusion and exclusion of parents and adults match that of the population survey with two additional restrictions applied to increase the homogeneity of the participants. While the population survey was open to adults of any age, the cohort study participants are 65 years of age or younger at the time of recruitment into the study. With comparatively little research available on the everyday experience of working aged adults, compared to older adults, the typical age of retirement was chosen as the upper limit. The participants in the cohort study are selected from one of three study sites in Canada: Manitoba, Ontario, or Atlantic Canada. Our intent is to recruit a total of 140 participants from the three study sites. The three regions represent different stages of development with regard to community and long term care, different population densities, and different ethnic and income groups. A third group of participants has been added; mature minors aged 13–16 whose parents are participants are providing data on their own behalf (see below).

#### Participant recruitment

The final page of the population survey provides respondents with information regarding the cohort study. Participants are invited to volunteer for the cohort study by providing their name and contact details on completion of the population survey. Participants expressing interest up to March 2012, and those who lived in one of the three geographic regions have been sequentially selected and invited to participate. Parents of children with neurological conditions are invited to participate with the knowledge that the impact on their family would be examined. If their child is a mature minor age 13–16, parents are asked during their second monthly interview if they believe their child is able and willing to participate in their own right. Mature minors are completing self-report surveys on three occasions; they are not participating in the monthly phone interviews). Transition from pediatric to adult services is known as a juncture at which many young people are lost to follow-up care. The inclusion of the age-specific tools will allow us to understand the variables of interest that are known to have unique features specific to this age group.

#### Ethics

Ethics approval/permission was granted from the same human research ethics bodies as the population survey. To include mature minors, additional consent procedures were required. The need for parental consent/assent to allow mature minors to participate in research varies across Canada; Nova Scotia requires authorization while other provinces in this study require parental consent. Consent forms developed were:

1. Parent/ guardian’s consent form for children age 13–16 years for provinces other than Nova Scotia,

2. Parent/guardian’s authorization form for children age 13–16 years in Nova Scotia.

3. Parent/guardian signature page for children under 18 years of age for all provinces.

#### Measurement tools

The broad variables examined in the cohort study include health status, participation, resource utilization, recent life events, self-management strategies, and support from health care, family and community resources. The outcome of resource utilization (family, health, social and community) is being monitored in more depth on a monthly basis using a semi-structured interview designed for the purpose. Planned and unplanned use of health, social and community resources are differentiated. To enrich the data on how the neurological condition has an impact on daily life, major changes in life circumstances (e.g. employment, living condition, family circumstances and health crises) are monitored. The population-based survey is being completed a second time by adults with neurological conditions and parents of children with a neurological condition, resulting in this data being collected at two data points. Additional data is being collected periodically using instruments not included in the population-based survey. For example, an additional tool for adolescents was added to the cohort study. GYV [28] is a youth-reported questionnaire that tells us what they think about the services they are receiving. Following semi-structured questions, interview guides include an open-ended question to allow participants to provide any additional information they would like to tell the research team that was not captured by the items. The instruments, along with the timeline for data collection for adults, parents and mature minors, are included in Table 2.

**Table 2:** Cohort study data collection instruments and timeline

	**Nov 2011**	**Dec 2011**	**Jan 2012**	**Feb 2012**	**Mar 2012**	**Apr 2012**	**May 2012**	**June 2012**	**July 2012**	**Aug 2012**	**Sept 2012**	**Oct 2012**	**Nov 2012**	**Dec 2012**
**Recruitment**	✓	✓	✓	✓	✓									
**Adult-NC Participants (Including Young Adult NC Participants)**														
Monthly interviews				1	2	3	4	5	6	7	8	9	10	11
The LINC Study Population Survey											1			
PACIC						1						2		
MSPSS					1				2				3	
Life-H					1				2				3	
GYV (Young Adults with NC Only)					1				2					3
**Parent Participants**														
Monthly Interviews				1	2	3	4	5	6	7	8	9	10	11
The LINC Study Population Survey											1			
PACIC						1						2		
MSPSS					1				2				3	
Life-H					1				2				3	
**Mature Minor Participants**														
Health Status					1				2					3
TRAQ					1				2					3
GYV					1				2					3
PAM					1				2					3
PACIC					1				2					3

#### Data collection procedures

Data is being collected via regularly scheduled 30–60 minute monthly phone calls by trained research assistants from February to December, 2012. While most data is being collected by phone interview, individuals have the option to complete additional surveys in the cohort study online or by paper and pencil. For those completing paper and pencil surveys, they receive a reminder phone call each month notifying them that the survey has been sent.

After every 10 minutes of interview time, research assistants ask participants if they wish to take a break. If a participant indicates that they would like to do so, the research assistant will stop and make arrangements to re-contact the participant at a time convenient to them.

#### Data analysis

We will use a combination of descriptive and inferential statistics to address the objectives of the cohort study. Data from the telephone interviews will be used to capture information that is either variable or difficult to recall, such as health providers seen, while data from surveys will be used to examine more stable constructs using validated tools.

We will first use descriptive statistics (means and standard deviations) to examine all participation, resource utilization, and health status outcome variables of interest as well as the potential predictors, such as self-efficacy and self-management strategies. Our analyses will seek to describe the distribution, prevalence, and rates of change across subgroups of the sample by demographic characteristics (age, gender, socioeconomic status, rural/remote) and condition (age of onset, degenerative, episodic). Analysis of other important characteristics identified in either the literature or our population survey will also be undertaken.

Once this basic descriptive analysis is complete and after checking the robustness of the data, multivariate analyses will be conducted to further describe variation in our three outcomes while controlling for confounding variables. General Linear Modeling (GLM) will be used to examine the variability in each outcome of interest over time and to control for inherent baseline variation in each outcome [39]. We anticipate using GLM to explore changes in mean scores, such as within-subject variation, for each outcome [39].

Multiple regression GLM procedures will also be used. GLM regression is a flexible method of data analysis typically used to examine the relationship between a dependent variable and an independent variable (or predictor variables) [40]. In the context of this analysis, the predictors include health status, self-efficacy, health care support, and self-management skills while the primary dependent outcome is on participation in everyday activities. If group differences in predictors exist, they will be incorporated into the regression models. Additionally, inherent group variation in initial participation scores will be controlled for in the regression models and the predictive contribution of independent variables will be assessed [41]. Our analyses will be guided by the research evidence and by what we find in our preliminary analyses. For example, we may examine rural/urban and diagnoses/co-morbidity differences in outcomes, and adjust for age-sex composition.

Qualitative data collected using open-ended questions during the telephone interviews will be analyzed using standard qualitative techniques as discussed in the following section describing the analysis to be used in the MPCS.

#### MPCS: Individual stories

The purpose of the MPCS is to capture and document the complexity of living with and managing a chronic condition. The MPCS will shed light on the capacity of health providers to support people to play an active part in their own care and engage in needed self-management tasks.

#### Participants and recruitment

Participants in the MPCS will be a sample of convenience, purposively selected from participants in the cohort study who express interest in continuing to be involved in the LINC study. The MPCS will recruit a total of eighteen focal participants. Twelve in-depth adult case studies will be conducted between May and August, 2012. Convenience and purposive sampling is being used to maximize variation on critical variables that are predicted to affect chronic disease self-management. In addition, six in-depth case studies involving parents of children as focal participants will be conducted over the same time period. Sampling is purposive to ensure we recruit cases that add to our depth of understanding and not necessarily to ensure representativeness. Where possible, mature minors with neurological conditions, aged 13–16 years, will be invited to participate along with their parents as focal participants. Collectively, these cases will represent the wide spectrum of characteristics of Canadians with neurological conditions. Our collective case is the everyday lived experience for persons with neurological conditions. We will explore the collective cases from multiple perspectives including the individual with the neurological condition (focal participant), and others who are influential in their daily life. Each focal participant will nominate between two and six individuals, ideally including at least one health provider and one family member for participation in the study.

#### Measurement tools

Four different semi-structured interview guides have been constructed for the various groups of participants: adult focal participants, parent focal participants, nominated support persons, and nominated healthcare providers. The focal participant interview guides (adult and parent) are longer and explore the areas of daily activities, the neurological conditions impact on life and relationships, self-management, experiences of healthcare and social services. The focal parent interview guide includes questions about parents’ perceptions of the impact on their child’s life and the perceived impact on their own life. The nominated interview guides (support persons and healthcare providers) are shorter and mainly concern support, care-giving, and healthcare experiences. The interview guides for the second interview with the focal participants are individually constructed with references from the first interview and the nominated interviews related to that participant.

The MPCS study design allows data collection through multiple strategies. Document review of care plans, program brochures, annual reports and policy documents prepared by health professionals and any others in the possession of and offered by focal participants, will be undertaken.

#### Data collection procedures

Focal participants will participate in two semi-structured face to face interviews, each of approximately 60–120 minutes. Interviews will take place at a time and in a location convenient to participants. The first interview will be more explorative in nature, whereas during the second interview the participant will be able to elaborate on topics from the first interview and topics raised by the nominated participants. During the second interview, participants will also be asked if they wish to have photos taken or a video recording made to be used in the knowledge translation phase of the project. Ethics approval was subsequently sought and granted to allow for this form of data collection. Nominated participants will participate in one interview of between 40 and 60 minutes duration. Following data analysis, we will perform member checks with the participants.

#### Data analysis

The data will be analyzed with an interpretive description approach [42]. Interpretive description is a qualitative approach useful for exploring questions of clinical relevance. In an interpretive description approach, a rigorous design is emphasized. This approach is suitable for large data materials, and data from different sources and multiple perspectives.

The steps of the analysis include initial reading and categorizing of the data in predefined areas (e.g., impact of illness, everyday life/participation, self-management, support and care-giving, healthcare and services experience). Those areas will then be further explored to identify common concerns, challenges or important issues. The analysis will both include case-by case and cross-case analyses. The analysis will be assisted using NVivo© 9.0, a software program designed to aid in conducting qualitative data analysis. Trustworthiness will be accomplished through triangulation of data sources, methods, and researchers. Rigor will be ensured by creating audit trails and supervision of research trainees.

Individual participant data from the three studies will be linked for analysis. Unique identification codes in the population survey will also be used in the cohort study and the MPCS in order to link data across the three components of the study. Parent data will also be linked to the data provided by their mature minors. Finally, focal participant data will be linked to that provided by the nominated participants in the MPCS. Participants will be informed that data will be linked.

## Discussion

The unique design of the LINC study will allow new insights but also poses limitations. With the nested design, participants in the MPCS will have participated in the cohort and population studies; the power is that data from all studies will be linked providing both depth and breadth of understanding across many diagnosis. This will be the first data of this kind collected in Canada. Use of non-diagnosis specific tools across a wide range of diagnosis is also unique with the potential to understand commonalities and differences by diagnosis, geographic area, age, severity of condition etc. Use of convenience sampling, bilingual formats and three forms of response (online, telephone and paper and pencil) increases access to people across Canada.

While representativeness of the sample will be hard to determine, this is will be the first data of its kind collected, providing a springboard for future research endeavors. Given that there are no mandatory reporting requirements for neurological conditions in Canada and that the true incidence and prevalence have not been confirmed, defining, locating and sampling a representative group of Canadians was not possible. Generalizability of results will therefore need to be done with caution.

There is mounting evidence that participants fill out paper and pencil, and online surveys similarly. In the majority of studies comparing the same versions of questionnaires, data collected with online and paper versions have been found to produce similar factor structures [43] and mean reliability coefficients, for example 0.91 vs. 0.89 respectively [44]. Additionally, online surveys are becoming increasingly popular in many areas [45]. This may be due to the better response rate, fewer missed responses and quicker return rate [46]. Furthermore online surveys offer lower overall costs, and more efficient distribution and collection [47]. Disadvantages can include an uncontrolled environment (e.g., random factors, distractions or the presence of others), a potential lack of anonymity and security, layout differences (due to software difference), and accessibility issues that question the generalizability of findings [47].

The LINC study will collect data using a variety of tools, some of which will be used together for the first time. Some of the standardized tools will be used for the first time in Canada or for the first time with people with neurological conditions. While the primary purpose of collecting the data is to answer the research questions, the potential to test the psychometric properties of the tools and or answer secondary questions will be high. In addition, the LINC study is part of a very large funding envelop that has funded approximately 15 major projects totaling over $15 million. While each project stands alone, all are examining neurological conditions in Canada in some way. The Public Health Agency of Canada has indicated that, in the event that it becomes obvious that sharing of de-identified data would benefit Canadians with neurological conditions, they would encourage this within the bounds of ethical research practice.

### Implications

The results of this study will inform service and policy development for people with neurological conditions and is expected to contribute to advances in service delivery and program development. An integrated approach to care is needed by people with neurological conditions. Understanding the types of services provided, the array of providers seeing patients, and more importantly the experiences of those receiving services represent shortcomings in our ability to understand both what impact the health care system is having on people’s experiences with chronic illness and what opportunities exist to improve chronic disease management [48,49].

## Abbreviations

LINC: The everyday experience of living with and managing a neurological condition; PALS: Participation and Activity Limitation Survey; NHCC: Neurological Health Charities of Canada; CIHI: Canadian Institute for Health Information; ICF: International Classification of Function; MPCS: Multiple Perspectives Case Studies; GSS: General Social Survey; CCHS: Canadian Community Health Survey; SLNCC: Survey of Living with a Neurological Condition; Life-H: Assessment of Life Habits; PedsQL: Pediatric Quality of Life; Neuro-QOL: Quality of Life Outcomes in Neurological Disorders; MSPSS: Multidimensional Scale of Perceived Social Support; GYV: Giving Youth a Voice; PIH: Partners in Health; PAM: Patient Activation Measure; PACIC: Patient Assessment of Chronic Illness; SLIQ: Simple Lifestyle Indicator Questionnaire; TRAQ: Transition Readiness Assessment Questionnaire; HUI: Health Utility Index; SF-36: Short-Form Health Status; ARDC: Atlantic Regional Data Centre; GLM: General Linear Modeling; NPHSNC: National Population Health Study of Neurological Conditions

## Competing interests

The authors declare that they have no competing interests.

## Authors’ contributions

TLP and JV conceived this study and led the LINC team in obtaining funding for the study. All authors contributed to the design and implementation of the study. JV, TP, KR and MV are contributing to the supervision of research assistants involved. LEW drafted the first version of this manuscript. All authors read and approved the final manuscript.

## Pre-publication history

The pre-publication history for this paper can be accessed here:

http://www.biomedcentral.com/1471-2377/13/30/prepub
